# Prevalence of SARS‐CoV‐2 Antibodies in Kosovo‐Wide Population‐Based Seroepidemiological Study

**DOI:** 10.1111/irv.70004

**Published:** 2024-09-03

**Authors:** Naser Ramadani, Sanije Hoxha‐Gashi, Dafina Gexha‐Bunjaku, Arijana Kalaveshi, Xhevat Jakupi, Isme Humolli, Aisling Vaughan, Richard Pebody, Pranvera Kacaniku‐Gunga, Violeta Jashari

**Affiliations:** ^1^ Faculty of Medicine University of Pristina “Hasan Pristina” Pristina Kosovo; ^2^ National Institute of Public Health Pristina Kosovo; ^3^ WHO Office in Pristina Pristina Kosovo; ^4^ WHO Regional Office for Europe Copenhagen Denmark

**Keywords:** antibodies, COVID‐19, Kosovo, SARS‐CoV‐2, seroepidemiological study, seroprevalence

## Abstract

**Background:**

Seroprevalence studies have proven to be an important tool in tracking the progression of the coronavirus disease 2019 (COVID‐19) pandemic. The aim of this study was to measure the seroprevalence of antibodies to severe acute respiratory syndrome coronavirus 2 (SARS‐CoV‐2) in the general population of Kosovo by gender, age group and region and among asymptomatic people.

**Method:**

The Institute of Public Health of Kosovo conducted a cross‐sectional population‐based survey, aligned with the protocols of the WHO Unity Studies, from the beginning of May to the end of June 2021.

**Results:**

The survey covered a total of 2204 people with a response rate of 91.8% (41.9% [923] males and 51.2% [1281] females). In May to June 2021, the prevalence of antibodies in the overall population (IgG antibodies ≥ 1.1) was 37.0%. Seroprevalence was 34.4% in men and 38.9% in women (*p* < 0.05), with the highest percentage (48.7%) found in the 60–69 years' age group. The overall prevalence of acute IgM antibodies (IgM ≥ 1.1) was 1% (95% CI: 0.7%–1.5%), with no significant difference between genders and the highest prevalence among participants of 60–69 years of age (1.6%; 95% CI: 0.7%–3.6%).

**Conclusion:**

A high prevalence of antibodies against SARS‐CoV‐2 was found in Kosovo before the start of the vaccination campaign. However, the results of the survey suggested that, by the end of June 2021, a desirable level of protection from the SARS‐CoV‐2 virus had not been reached.

## 1 Introduction

After its detection in Wuhan, Hubei Province, China, in December 2019 [1, 2], the severe acute respiratory syndrome coronavirus 2 (SARS‐CoV‐2) spread rapidly around the world [3]. It was declared a public health emergency of international concern on 30 January 2020 [4]. Globally, as of 11 April 2023, 762,791,152 confirmed cases of COVID‐19, including 6,897,025 deaths, had been reported to the World Health Organization (WHO) [5].

Kosovo, located in southeast Europe with a population of 1.78 million [6] (61.7% rural, 38.3% urban), encountered various challenges in dealing with the SARS‐CoV‐2 virus, which were exacerbated by the fragility of its health systems in facing this global challenge.

The first cases of COVID‐19 were reported in Kosovo on 13 March 2020, involving a 77‐year‐old man from Vitia who had recently returned from Italy and an Italian woman in her early 20s [7]. In response to the epidemiological situation, the health authorities in Kosovo declared a public health emergency and introduced public health and social measures, including closure of the schools and restrictions on international travel (including quarantine and testing) and movement up to 31 May 2020.

Between 13 March 2020 and 31 June 2021, 107,768 cases of COVID‐19 were confirmed in Kosovo, including 2258 deaths, according to an analysis conducted by the Institute of Public Health of Kosovo and the WHO [8]. The COVID‐19 vaccination programme started on 29 March 2021 with the rollout of vaccinations for health‐care workers and people > 65 years, but mass vaccination started on 15 June 2021 [9]. Between 29 March and 15 June 2021, 99,000 doses of vaccine were administered [10], resulting in the vaccination of around 5% of population. Initially, the necessary quantities of vaccines were secured: 1.2 million vaccines received through a bilateral contract from Pfizer, donation of vaccines for 20% of the population received through COVAX and other donations.

Seroprevalence studies have proven to be an important tool for monitoring the progression of the COVID‐19 pandemic and providing estimates of the true burden of disease. Despite a significant number of seroprevalence studies having been conducted globally [11], there is a paucity of seroprevalence studies after several waves of the pandemic, and before mass vaccination, in Central and Eastern Europe [12, 13], especially for the low‐ and middle‐income economies of the WHO European Region.

In 2021, 1 year into the pandemic, the Institute of Public Health of Kosovo conducted a Kosovo‐wide seroprevalence study. The aim was to gain an understanding of the true population exposure to the virus prior to mass vaccination, as well as the proportion of asymptomatic infections in the population, and to inform public health strategies to manage emergencies with infective diseases.

## 2 Methods

The Institute of Public Health of Kosovo conducted a cross‐sectional population‐based seroprevalence survey, aligned with the protocols of the WHO Unity Studies, in Kosovo, from the beginning of May to the end of June 2021 regardless of waves, with technical support from the WHO [14]. During this period in Kosovo, 2560 positive cases of COVID‐19 were reported, with 84 deaths.

### 2.1 Study Design and Population Recruitment

The survey was carried out using multistage, age‐stratified population sampling. During the first stage, the population was stratified according to the 34 municipalities of Kosovo; during the second stage, each health region was stratified by age group (2–9, 10–19, 20–29, 30–39, 40–49, 50–59, 60–69 and 70+ years). According to the data of the Kosovo Agency of Statistics (KAS) 38.1% of the population of Kosovo are 0–19 years old, 32.1% are 20–39 years old, 20.1% are 40–59 years old, and 9.8% are 60 or more years old [6]. The participants were selected from the households sampling list generated randomly by the KAS. One individual per household (defined as a group of people—two or more—living in the same residence) was invited to participate in the serosurvey, selected using Kish method. All individuals identified for recruitment, irrespective of age and acute or prior SARS‐CoV‐2 infection, were included in the study. Refusal to give informed consent and contraindication to venipuncture were exclusion criteria.

### 2.2 Data Collection and Laboratory Testing

The survey was conducted in seven sites: the Institute of Public Health and six regional centres of public health. Informed consent was obtained from all individuals willing to participate in the investigation prior to the performance of any procedure. Consent for the participation of children (< 18 years old) was obtained from the parent/caregiver. Each participant was randomly recruited and requested to complete an epidemiological questionnaire, covering demographic, clinical and exposure‐related information. Blood samples were collected from adults and children (5 and 2.5 mL, respectively) through venipuncture by trained staff and transported at 4.0°C to the Department of Microbiology of the Institute of Public Health, where they were aliquoted and stored at −80°C. In addition, nasopharyngeal swabs were collected for testing for SARS‐CoV‐2 infection by real‐time PCR (RT‐PCR). All laboratory investigations were conducted at the Department of Microbiology.

Serum samples were analysed for the presence of specific IgM and IgG antibodies against SARS‐CoV‐2 using Chorus SARS‐CoV‐2 IgG and Chorus SARS‐CoV‐2 IgM tests. Laboratory analyses to detect the levels of IgM and IgG antibodies were performed separately. According to DIESSE, the sensitivity of the Chorus SARS‐CoV‐2 IgG test was 100.0% (95% CI: 98.2–100.0), and specificity was 99.6% (95% CI: 97.9–99.9). In the case of the Chorus SARS‐CoV‐2 IgM test, sensitivity was 87.7% (95% CI: 77.5–93.6), and specificity 98.1% (95% CI: 96.6–99.0).

All samples that were positive to the presence of antibodies against SARS‐CoV‐2 in the qualitative tests were additionally tested with DIESSE's Chorus SARS‐CoV‐2 ‘neutralizing’ antibody test for the presence of neutralizing antibodies (sensitivity: 99.6% [95% CI: 97.7–99.9]; specificity: 99.8% [95% CI: 99.2–99.9]) [15].

### 2.3 Ethical Considerations

The study protocol was approved by the Doctors' Chamber Ethical Committee in Kosovo (reference numbers 20/2020 and 24/2021) and the WHO Research Ethics Review Committee (protocol ID: CERC.0013E).

### 2.4 Statistical Analysis

A sample size of 2400 was estimated based on an expected seroprevalence of 50% and a margin of error of 0.5%, and 300 samples were needed for each age group (2–9, 10–19, 20–29, 30–39, 40–49, 50–59, 60–69 and 70+ years).

The data analysis was conducted in Epi Info, Version 3.5.1, developed by the Centers for Disease Control and Prevention, Atlanta, Georgia, USA, and the prevalence rates with corresponding 95% CIs were estimated. The results were considered statistically significant if *p* < 0.05.

## 3 Results

**FIGURE 1:**
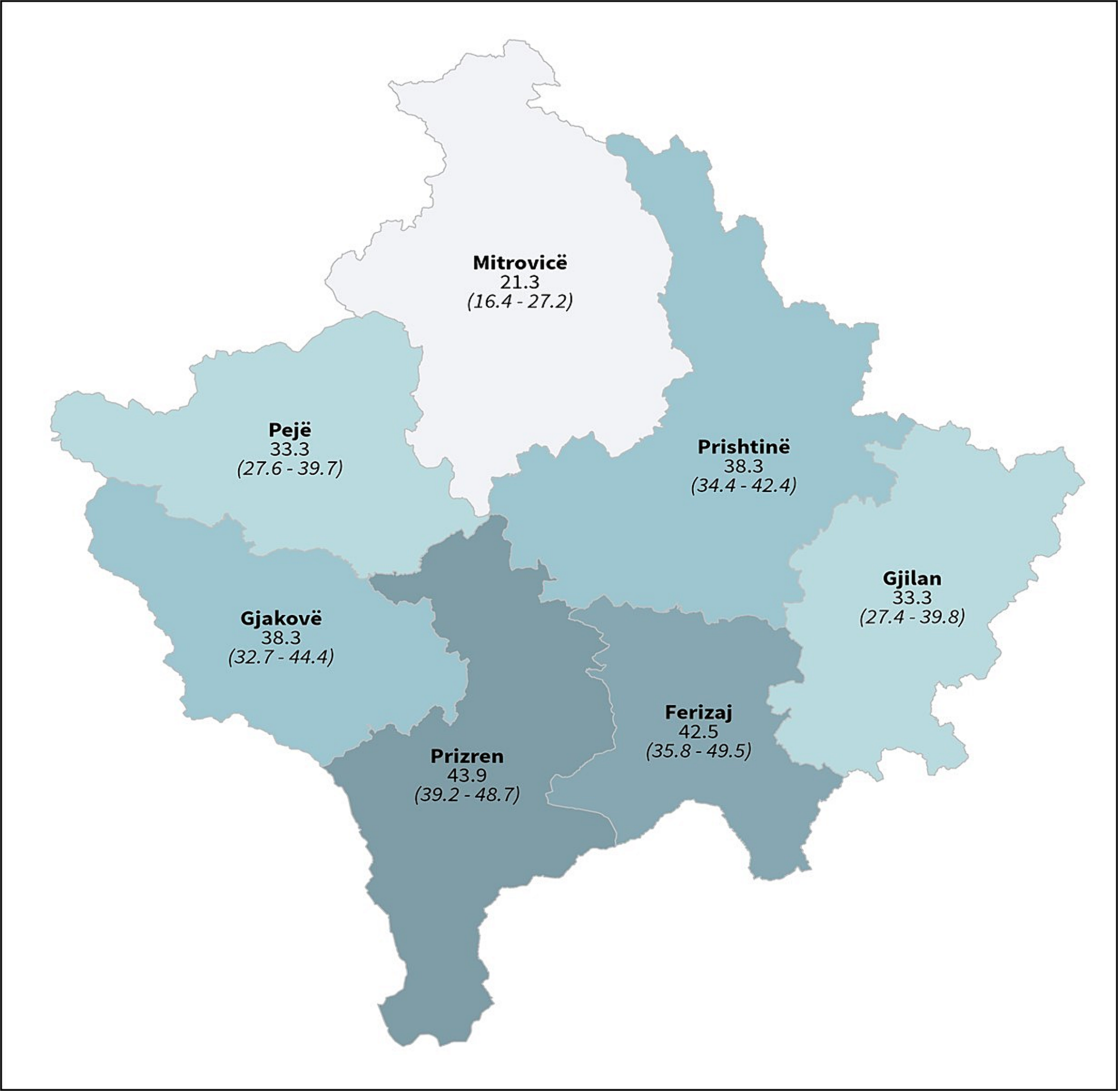
Geographical distribution of SARS‐CoV‐2 seroprevalence in Kosovo, May–June 2021. The designations employed and the presentation of the material in this publication do not imply the expression of any opinion whatsoever on the part of WHO concerning the legal status of any country, territory, city or area or of its authorities or concerning the delimitation of its frontiers or boundaries. All references to Kosovo in this document should be understood to be in the context of the United Nations Security Council Resolution 1244 (1999).

Of the 2400 individuals invited to participate in the study from May to June 2021, 2204 (91.8%) did so, including 923 males (41.9%) and 1281 females (51.2%). Most of the participants (1854 or 84.1%) were between 20 and 70 years old, those in the younger (2–19 years; 201 or 9.2%) and older (70+ years; 149 or 6.8%) age groups representing the lowest numbers (Table 1). Coverage did not vary significantly by region, the highest being in Gjakova (100%) and the lowest in Ferizaj (84.7%) (Table 1 and Figure 1). The overall prevalence of IgG antibodies was 37.0% (95% CI: 35.0–39.1). In men, the prevalence was 34.4% (95% CI: 31.4–37.6) and in women 38.9% (95% CI: 36.2–41.6). Seropositivity was found in all age groups, the trend increasing by age group. The highest level of seropositivity was observed in the age group of 60–69 years (48.7%; 95% Cl: 43.6–53.9), followed by 70+ years (47.3%; 95% CI: 39.2–55.4), 50–59 years (39.4%; 95% Cl: 34.8–44.1), 40–49 years (37.2%; 95% Cl: 32.6–42.0), 10–19 years (38.1%; 95% Cl: 30.9–45.9), 30–39 years (27.2%; 95% Cl: 22.6–32.3), 20–29 years (24.7%; 95% Cl: 20.0–30.1) and 1–9 years (25.0%; 95% Cl: 12.7–43.4). Seroprevalence varied by region, ranging from 43.9% (95% Cl: 39.2–48.7) in the Prizren region to 21.3% (16.4–27.2) in the Mitrovica region (Table 2). The overall prevalence of IgM antibodies (which is indicative of the acute phase of disease) was 1% (95% CI: 0.7–1.5) (Table 2) with no significant difference between the genders: The prevalence of IgM in men was 1.1% (95% CI: 0.6–2), but in women it was 0.9% (95% CI: 0.5–1.6). IgM seropositivity was higher among participants over 20 years old, mainly in the region of Gjilan where it was recorded as 3.5% (95% Cl: 1.8–6.9) (Table 2).

**TABLE 1:** Participant demographics.

	*N*	%
Total	2204	100.0
Age group (years)		
2–9	32	1.5
10–19	169	7.7
20–29	294	13.3
30–39	329	14.9
40–49	427	19.4
50–59	437	19.8
60–69	367	16.7
70+	149	6.8
Sex		
Male	923	41.9
Female	1281	51.2
Region		
Ferizaj	211	9.6
Gjakovë	272	12.3
Gjilan	230	10.4
Mitrovicë	231	10.5
Pejë	240	10.9
Pristinë	589	26.7
Prizren	431	19.6
Previous SARS‐CoV‐2 infection ^a^		
Yes	615	27.9
No	1589	72.1
Number of previous infections		
1	367	16.7
2	160	7.3
3	57	2.6
4	17	0.8
5	6	0.3
6+	8	0.4
PCR confirmation (*n* = 615)	229	37.2
Symptoms in last 6 months	413	18.7
Medical attention sought	380	17.2
School or work absenteeism	114	5.2
Hospitalized	31	1.4
Self‐declared^a^		

^a^ Previous SARS CoV‐2 infection was self declared from participants in the study.

**TABLE 2:** Prevalence of SARS‐CoV2 antibodies (IgG and IgM) by sex, age group and region.

		Prevalence of IgG ≥ 1.1		Prevalence of IgM ≥ 1.1	
	*N*	*n* (%)	95% CI	*n* (%)	95% CI
	2204	816 (37.0)	35.0–39.1	22 (1.0)	0.7–1.5
Sex					
Men	923	318 (34.4)	31.4–7.6	10 (1.1)	0.6–2.0
Women	1281	498 (38.9)	36.2–41.6	12 (0.9)	0.5–1.6
Age group (years)					
1–9	32	8 (25.0)	12.7–43.4	0 (0.0)	0.0–0.0
10–19	169	64 (38.1)	30.9–45.9	0 (0.0)	0.0–0.0
20–29	294	73 (24.7)	20.0–30.1	4 (1.4)	0.5–3.6
30–39	329	89 (27.2)	22.6–32.3	3 (0.9)	0.3–2.8
40–49	427	160 (37.2)	32.6–42.0	5 (1.2)	0.5–2.8
50–59	437	173 (39.4)	34.8–33.1	3 (0.7)	0.2–2.1
60–69	367	179 (48.7)	43.6–53.9	6 (1.6)	0.7–3.6
70+	149	70 (47.3)	39.2–55.4	1 (0.7)	0.1–4.7
Region					
Ferizaj	211	90 (42.5)	35.8–49.5	0 (0.0)	0.0–0.0
Gjakovë	272	104 (38.3)	32.7–44.4	2 (0.7)	0.2–2.9
Gjilan	230	77 (33.3)	27.4–39.8	8 (3.5)	1.8–6.9
Mitrovicë	231	49 (21.3)	16.4–27.2	3 (1.3)	0.4–4.0
Pejë	240	80 (33.3)	27.6–9.7	0 (0.0)	0.0–0.0
Prishtinë	589	226 (38.3)	34.4–42.4	3 (0.5)	0.2–1.6
Prizren	431	190 (43.9)	39.2–48.7	6 (1.4)	0.6–3.1

Participants were tested for SARS‐CoV‐2 by RT‐PCR (see Table S1). At the time of sampling, the overall PCR positivity was 0.0 (95% Cl: 0.0–0.3). There was only one positive case of women aged 60–69 years from the region of Prizren.

Of the 2204 participants, 1121 (50.9%) were asymptomatic. From asymptomatic people (*n* = 1121), 370 or 33.1% (95% Cl: 30.4–36.0) were found to be IgG seropositive. From asymptomatic men (*n* = 526), 164 or 31.3% (95% Cl: 27.4–35.5) IgG seropositive, and from asymptomatic women (*n* = 595), 206 or 34.7% (95% Cl: 30.9–38.8) were IgG seropositive. By region, the highest level of seroprevalence among asymptomatic people was registered in Prizren (38.2%) (95% Cl: 32.5–44.2), and the lowest (18.0%) (95% Cl: 9.5–31.5) in Peja region (Table 3).

**TABLE 3:** Prevalence of past COVID‐19 infection (IgG ≥ 1.1) among asymptomatic people.

Prevalence among asymptomatic people			
	*N*	*n* (%)	95% CI
	1121	370 (33.1)	30.4–36.0
Sex			
Men	526	164 (31.3)	27.4–35.5
Women	595	206 (34.7)	30.9–38.8
Age group (years)			
1–9	27	7 (25.9)	12.4–46.3
10–19	121	49 (40.4)	31.7–49.7
20–29	167	39 (23.3)	17.3–30.5
30–39	179	43 (24.3)	18.4–31.3
40–49	205	67 (32.7)	26.4–39.6
50–59	191	66 (34.8)	28.3–42.0
60–69	171	75 (43.8)	36.4–51.4
70+	60	24 (40.7)	28.7–53.8
Region			
Ferizaj	37	11 (28.6)	15.8–46.1
Gjakovë	76	21 (27.8)	18.5–39.4
Gjilan	144	43 (29.9)	22.8–38.2
Mitrovicë	88	18 (20.9)	13.5–31.0
Pejë	54	10 (18.0)	9.5–31.5
Pristinë	449	163 (36.4)	32.0–41.1
Prizren	273	104 (38.2)	32.5–44.2

## 4 Discussion

This was the first Kosovo‐wide cross‐sectional SARS‐CoV‐2 seroprevalence investigation to be carried out. By May–June 2021, 37.0% (95% CI: 35.0–39.1) of the population had been exposed to SARS‐CoV‐2. The seroprevalence varied by geography, gender and age group, which was consistent with the general picture from the broader surveillance system. Kosovo is a small country with 10,887 km^2^ with a population density of 177.4 inhabitants per km^2^ [6]. The high prevalence in Prizren compared to other regions of Kosovo can be attributed to its proximity to Albania. In Albania, seroprevalence was 59.4% during July to August 2021 [16]. In one systematic review in 2021, Ya'qoub et al. [17] describe sex and gender differences in a lot of studies. These differences are likely attributed to a combination of factors like hormonal differences, immune response and differences in attitudes and behaviors.

Several large seroprevalence studies had been conducted elsewhere in the WHO European Region with varying results [18]. In their systematic review, Bergeri et al. showed that, by June 2021, the overall seroprevalence in low‐ and middle‐income economies in the WHO European Region was 48.7% (47.7%–49.7%), compared to 22.4% (21.1%–23.8%) in July 2020 [16]. A nationwide study of the general population in Croatia, performed after the first (May–July 2020) and second (December 2020–February 2021) waves of the pandemic, found a significant difference in the overall seroprevalence rate over time (IgG 2.2%–25.1%) [13]. A study conducted in Tirana, Albania, found that the proportion of individuals classified as seropositive during the first round of the pandemic in early July 2020 was 7.5% (95% CI: 4.3%–10.7%). This proportion rose sharply in the second round, reaching 48.2% (95% CI: 44.8%–51.7%) by late December 2020 [12]. In Saint Petersburg, Russia, the adjusted seroprevalence was 9.7% (95% CI: 7.7–11.7) in May–June 2020, 13.3% (95% CI: 9.9–16.6) in July–August 2020, 22.9% (95% CI: 20.3–25.5) in October–December 2021 and 43.9% (95%CI: 39.7–48.0) in February–April 2021 [19]. National or equivalent representative seroprevalence surveys conducted in other countries, territories and areas have shown similar variable seroprevalence estimates [20, 21].

Initially, surveillance in Kosovo focused primarily on patients and contacts with symptoms, with more than half of the confirmed cases showing symptoms and the remaining cases being asymptomatic. In addition, the role of pre‐symptomatic, asymptomatic or subclinical infections in the human‐to‐human transmission of SARS‐CoV‐2 virus is not well understood. With a novel coronavirus, initial seroprevalence in the population is assumed to be negligible. Therefore, the surveillance of antibody seropositivity in a population could allow for inferences about the extent of infection and its cumulative incidence in the population. For the survey under discussion, a standardized WHO protocol was used [14]. This enabled the systematic collection and rapid sharing of epidemiological exposure data and biological samples in a format that could easily be aggregated, tabulated and analysed across many different settings globally. These timely estimates of SARS‐CoV‐2 and information about its severity and attack rates made it possible to inform public health responses and policy decisions, which is particularly important in the context of a novel respiratory pathogen, such as SARS‐CoV‐2. Limitation of this study was only that The COVID‐19 vaccination programme started on 29 March 2021 with the rollout of vaccinations for health‐care workers and people > 65 years, but mass vaccination started on 15 June 2021 but the exclusion criterion from study was if somebody was vaccinated. Such studies have been performed in many health systems. Some of them have been limited to specific geographical areas or carried out on non‐representative samples of the population, but surveys designed as national or equivalent representative surveys have found substantial geographical variability with higher seroprevalence in more densely populated areas [22, 23, 24, 25, 26, 27, 28, 29, 30, 31, 32].

## 5 Conclusions

This serological survey demonstrated that SARS‐CoV‐2 was likely more widespread in Kosovo than indicated by the number of cases reported in June 2021. However, its findings showed that the population of Kosovo had not reached a suitable level of protection from the SARS‐CoV‐2 virus in the beginning of May to the end of June 2021. Regular seroprevalence studies are required to monitor population seroprevalence, assess risk factors and inform targeted public health policies.

## Author Contributions

**Naser Ramadani:** conceptualization, data curation, formal analysis, funding acquisition, investigation, methodology, project administration, resources, software, supervision, validation, visualization, writing – original draft, writing – review and editing. **Sanije Hoxha‐Gashi:** conceptualization, data curation, formal analysis, funding acquisition, investigation, methodology, project administration, resources, software, supervision, validation, visualization, writing – original draft, writing – review and editing. **Dafina Gexha‐Bunjaku:** conceptualization, data curation, formal analysis, funding acquisition, investigation, methodology, project administration, resources, software, supervision, validation, visualization, writing – original draft, writing – review and editing. **Arijana Kalaveshi:** conceptualization, data curation, formal analysis, funding acquisition, investigation, methodology, project administration, resources, software, supervision, validation, visualization, writing – original draft, writing – review and editing. **Xhevat Jakupi:** conceptualization, data curation, formal analysis, funding acquisition, investigation, methodology, project administration, resources, software, supervision, validation, visualization, writing – original draft, writing – review and editing. **Isme Humolli:** conceptualization, data curation, formal analysis, funding acquisition, investigation, methodology, project administration, resources, software, supervision, validation, visualization, writing – original draft, writing – review and editing. **Aisling Vaughan:** conceptualization, data curation, formal analysis, funding acquisition, investigation, methodology, project administration, resources, software, supervision, validation, visualization, writing – original draft, writing – review and editing. **Richard Pebody:** conceptualization, data curation, formal analysis, funding acquisition, investigation, methodology, project administration, resources, software, supervision, validation, visualization, writing – original draft, writing – review and editing. **Pranvera Kacaniku‐Gunga:** conceptualization, data curation, formal analysis, funding acquisition, investigation, methodology, project administration, resources, software, supervision, validation, visualization, writing – original draft, writing – review and editing. **Violeta Jashari:** conceptualization, data curation, formal analysis, funding acquisition, investigation, methodology, project administration, resources, software, supervision, validation, visualization, writing – original draft, writing – review and editing.

## Conflicts of Interest

The authors declare no conflicts of interest.

## Supporting information


**Table S1** Prevalence of viral RT‐PCR positivity by sex, age group and region.

## Data Availability

The data that support the findings of this study are available on request from the corresponding author.
