# Supplementary material for: Prevalence of SARS‐CoV‐2 Antibodies in Kosovo‐Wide Population‐Based Seroepidemiological Study
Source: Influenza Other Respir Viruses. 2024 Sep 3;18(9):e70004. doi: 10.1111/irv.70004 (PMC11369638; doi:10.1111/irv.70004)
Supplement: Supplementary file 1 — Table S1 Prevalence of viral RT‐PCR positivity by sex, age group and region. [file IRV-18-e70004-s001.docx]

**Supplementary material**

**Supplementary Table 1. Prevalence of viral RT-PCR positivity by sex, age group and region**

| **RT PCR positivity** | | | |
| --- | --- | --- | --- |
| **N** | | **n (%)** | **95% CI** |
|  | 2204 | 1 (0.0) | 0.0–0.3 |
| **Sex** | | | |
| Men | 923 | 0 (0.0) | 0.0–0.0 |
| Women | 1281 | 1 (0.1) | 0.0–0.6 |
| **Age group (yr)** | | | |
| 1–9 | 32 | 0 (0.0) | 0.0–0.0 |
| 10–19 | 169 | 0 (0.0) | 0.0–0.0 |
| 20–29 | 294 | 0 (0.0) | 0.0–0.0 |
| 30–39 | 329 | 0 (0.0) | 0.0–0.0 |
| 40–49 | 427 | 0 (0.0) | 0.0–0.0 |
| 50–59 | 437 | 0 (0.0) | 0.0–0.0 |
| 60–69 | 367 | 1 (0.3) | 0.0–1.9 |
| 70+ | 149 | 0 (0.0) | 0.0–0.0 |
| **Region** | | | |
| Ferizaj | 211 | 0 (0.0) | 0.0–0.0 |
| Gjakovë | 272 | 0 (0.0) | 0.0–0.0 |
| Gjilan | 230 | 0 (0.0) | 0.0–0.0 |
| Mitrovicë | 231 | 0 (0.0) | 0.0–0.0 |
| Pejë | 240 | 0 (0.0) | 0.0–0.0 |
| Pristina | 589 | 0 (0.0) | 0.0–0.0 |
| Prizren | 431 | 1 (0.2) | 0.0–1.6 |
